# Role of Fiber Shaft Length in Tumor Targeting with Ad5/3 Vectors

**DOI:** 10.3390/genes13112056

**Published:** 2022-11-07

**Authors:** Maximilian Richter, Hongjie Wang, André Lieber

**Affiliations:** Department of Medicine, Division of Medical Genetics, University of Washington, Seattle, WA 98195, USA

**Keywords:** oncolytic virus, serotype 3, desmoglein 2, coagulation factor X

## Abstract

Desmoglein 2 (DSG2) is overexpressed in many epithelial cancers and therefore represents a target receptor for oncolytic viruses, including Ad5/3-based viruses. For most Ad serotypes, the receptor-binding fiber is composed of tail, shaft, and knob domains. Here, we investigated the role of the fiber shaft in Ad5/3 tumor transduction in vitro and in human DSG2-transgenic mice carrying human DSG2^high^ tumors. DSG2tg mice express DSG2 in a pattern similar to humans. We constructed Ad5/3L (with the “long” Ad5 shaft) and Ad5/3S (with the “short” Ad3 shaft) expressing GFP or luciferase. In in vitro studies we found that coagulation factor X, which is known to mediate undesired hepatocyte transduction of Ad5, enhances the transduction of Ad5/3(L), but not the transduction of Ad5/3(S). We therefore hypothesized that Ad5/3(S) would target DSG2^high^ tumors while sparing the liver after intravenous injection. In vivo imaging studies for luciferase and analysis of luciferase activity in isolated organs, showed that Ad5/3(L) vectors efficiently transduced DSG2^high^ tumors and liver but not normal epithelial tissues after intravenous injection. Ad5/3(S) showed minimal liver transduction, however it failed to transduce DSG2^high^ tumors. Further modifications of the Ad5/3(S) capsid are required to compensate for the lower infectivity of Ad5/3(S) vectors.

## 1. Introduction

Currently, there are more than 104 human adenoviruses (Ads) in seven species (A to G) [1,2]. For most Ads, infection involves the high affinity/avidity binding of the fiber knob to a cellular attachment receptor followed by a second interaction between the viral penton base with cellular integrins, which triggers the internalization of attached virions. A subgroup of species B Ads, including serotype 3 (Ad3), uses desmoglein 2 (DSG2) as a receptor [3]. DSG2 is best known as an integral component of epithelial junctions [4]. DSG2 is overexpressed and a predictor of poor prognosis in multiple types of cancer, including skin [5,6], non-small cell lung cancer [7], lung adenocarcinoma [8,9], hepatocellular carcinoma [10], ovarian cancer [11], and gastric cancer [12], indicating that tumors take advantage of DSG2 overexpression as a means of forming tight physical barriers and contributing to resistance against therapeutics [13,14]. Based on this, DSG2-interacting Ad vectors were developed for oncolytic virus therapy. For more than a decade, chimeric Ad vectors that possess the serotype 5 (Ad5) fiber shaft and Ad3 knob (Ad5/3), have been used for cancer therapy in humans with a very good efficacy and an acceptable safety profile after intravenous injection [15,16,17,18]. More recently, clinical studies with oncolytic vectors based completely on Ad3 have been performed [19].

The fiber knob largely determines the receptor tropism of adenoviruses. In the case of Ad5/3 vectors, the substitution of the Ad5 knob by the Ad3 knob domain switches the tropism of the vector from the Coxsackie-adenovirus receptor (CAR) to DSG2. However, among the human Ad serotypes with different tissue tropisms, not only the knob, but also the length of the fiber shaft domain, varies significantly. The rod-like fiber shaft contains repeats of up to 14 amino acids forming β sheets, with the number of repeats ranging from 6 (in Ad3, Ad11, and Ad35) to 23 (in Ad12) [20]. Ad3 has six shaft motifs and a length of ~7 nm. Ad5 has 22 motifs and a length of 37 nm. The length of the fiber shaft has to be seen in the context of the knob and penton binding to cells. Electron microscopy studies of Ad5 fibers revealed a bend in the fiber shaft that is thought to be facilitated by a four amino acid (KKTK) insertion into the short linker between β-strands of the third β-repeat It is thought that the flexibility of the Ad5 shaft allows for the simultaneous binding of CAR by the fiber knob and multiple αv integrins by the Ad5 penton base [21]. In contrast, for the Ad3 fiber, the insertion of two residues in the third repeat is not sufficient for fiber flexibility [22,23]. It is thought that for Ad3 infection, DSG2 is flexible enough, so it can interact with the Ad3 fiber knob [24]. Notably, CryoEM studies of the Ad3 fiber knob–DSG2 complex reveals a unique stoichiometry of 1:1 and 2:1 (DSG2: knob trimer), not previously observed for other adenovirus–receptor (e.g., CAR and CD46) complexes [25].

The nature of the high-affinity binding receptor can affect the infection of long (Ad5) and short (Ad3 or Ad35)-shafted vectors. Studies with Ad5 vectors containing the CAR-binding Ad5 fiber knob and either the native (long) Ad5 fiber shaft or the (short) Ad3 fiber shaft showed that infectivity of the short-shafted Ad5 vectors was 20- or 4-fold less by plaque assay or GFP expression in 293 cells, respectively [26]. However, no differences in in vitro infectivity were observed for short- or long-shafted, CD46-interacting Ad5/35 vectors [26]. The influence of the fiber shaft length on infection of DSG2-targeting Ad5/3 vectors has not been studied so far.

Previously, we found that intravenously injected short-shafted Ad5/35 vectors did not efficiently transduce liver but targeted metastatic tumors that express CD46 at levels comparable to human xenograft tumors [26]. Along this line, no efficient liver transduction with short-shafted Ad5/35 vectors was found in baboons after intravenous injection [27]. The findings that short-shafted Ad5 and Ad5/35 vectors have reduced liver transduction was confirmed by a number of other groups [28,29,30]. In contrast, intravenous injection of Ad5/35 vectors containing the long Ad5 fiber shaft resulted in undesired transduction of hepatocytes in mice [31]. Subsequent studies found that hepatocyte transduction of Ad5 vectors was mediated by vitamin K-dependent blood coagulation factors that bind to the Ad5 hexon [31,32].

Based on the findings with short-shafted Ad5/35 vectors, we hypothesized that the use of short-shafted, DSG2-targeting Ad5/3 vectors would efficiently transduce DSG2^high^ tumors while sparing the liver after intravenous injection into an adequate human DSG2-transgenic (DSG2tg) mouse model. This hypothesis is, in part, supported by observations that oncolytic vectors completely derived from Ad3 (and containing the original short Ad3 fiber shaft) displayed remarkable anti-tumor efficacy and good safety [19,33,34].

## 2. Materials and Methods

***Recombinant proteins:*** Recombinant human DSG2 protein was from Leinco Technologies, Inc. (St. Louis, MO, USA). The recombinant Ad3 fiber knob (JO4) was produced in *Escherichia coli* with N-terminal 6-His tags using the pQE30 expression vector (Qiagen, Valencia, CA, USA) and purified by nickel–nitrilotriacetic acid agarose chromatography as described elsewhere [35]. Human factor X was from Haematologic Technologies (Essex Junction, VT, USA) (HCX-0050, lot T1206).

***Adenovirus vectors:*** The construction of Ad5/3L-GFP and Ad5/3S-GFP was described earlier [36]. Viral particle (VP) concentrations were determined spectrophotometrically by measuring the optical density at 260 nm (OD260). Titers of plaque-forming units (pfu) were performed using 293 cells as described elsewhere [35]. The VP/PFU ratio was 20:1 for all virus preparations.

***Cells:*** Colon cancer T84 cells (ATCC CCL-248) were cultured in a 1:1 mixture of Ham’s F12 medium and DMEM, 10% FBS, Glu, and Pen-Strep. To achieve cell polarization, 2 × 10^5^ T84 cells were cultured in 6.5-mm Transwell inserts (0.4-μm pore size) (Costar Transwell Clears, Corning, New York, NY, USA) for more than 20 days until transepithelial resistance was stable. To create TC1-hDSG2 cells, TC1 cells were transduced with a lentivirus vector expressing the human DSG2 cDNA under the control of the EF1α promoter [3]. hDSG2^high^ cells were isolated by a fluorescence-activated cell sorter (FACS) one week later. CHO-K1 (heparan sulfate proteoglycan [HSPG] expressing cells; ATCC CCL-61) and CHO-E606 (HSPG-negative cells; ATCC CCL-61) were from the American Type Culture Collection. CHO cells were cultured in a minimal essential medium (MEM) supplemented with 10% FBS, 200 mM asparagine, and 200 mM proline.

***Luciferase assay:*** Luciferase activity in cell and tissue lysates was measured using the Promega luciferase assay kit in a 96-well plate luminometer. Cells were lysed using the reagent lysis buffer provided by the manufacturer. Tissue homogenates were submitted to three freeze–thaw cycles and then centrifuged. For all organs, 20 μL of lysate were used per well for measurement of luciferase and total protein.

***Competition of Ad5/3-GFP(S) and Ad5/3-GFP(L) by recombinant Ad3 fiber knob (JO4) or rDSG2.*** Colon cancer T84 cells were incubated with 20 μg/mL of the proteins for 1 h before viruses were added at a multiplicity of infection (MOI) of 150 pfu/cell. After 1 h of infection, the medium was replaced and GFP expression was analyzed 20 h later.

***In vitro transduction studies with Factor X.*** The day before infection, 3 × 10^5^ CHO cells were seeded per well (24-well plate). The next day, attached cells were counted and virus was added at the indicated MOI in 1 mL of growth medium containing FCS either with or without human factor X (8 μg/mL). After 2 h of incubation with Ad vectors, the medium was removed, and cells were washed. Luciferase was measured after 24 h of incubation in complete medium.

***Analysis of intracellular trafficking.*** The workflow to measure attachment, internalization, and nuclear transport of viral particles is shown in Figure S1. A Nuclear Extraction Kit from abcam (ab113474) (Cambridge, UK) was used. Virions were measured based on viral genomes, which were quantified by qPCR as follows: Total genomic DNA (gDNA) was extracted by using Quick-DNA miniprep kit (Zymo Research, Irvine, CA, USA) following the manufacturer’s instructions. gDNA samples were analyzed for vector copy numbers (VCN) with the following primers: GFP forward: TCGTGACCACCCTGACCTAC, GFP reverse: GGTCTTGTAGTTGCCGTCGT, human GAPDH forward: caaattccatggcaccgtca, human GAPDH reverse: tcctagttgcctccccaaag. qPCR was performed using the Power SYBR Green PCR Master Mix (Thermo Fisher Scientific, Waltham, MA, USA). Each reaction was run in triplicate. Serial dilutions of purified Ad5/3-GFP viral DNA diluted in gDNA extracted from control cells were used for a standard curve.

***Immunofluorescence analysis of TC1-DSG2 tumor sections***: Frozen OCT sections were fixed with methanol-acetone (1:1) and incubated with antibodies: rabbit claudin 7 (abcam), mouse monoclonal antibody anti-DSG2 (clone 6D8; Cell Sciences, Canton, MA), rabbit CD31 (abcam), rabbit anti-laminin (abcam). Images were taken by a Leica DMLB microscope (Wetzlar, Germany), using a Leica DFC300FX digital camera and Leica Application Suite version 2.4.1 R1 software (Leica, Heerbrugg, Germany).

***Animal studies.*** These studies were carried out in strict accordance with the recommendations in the Guide for the Care and Use of Laboratory Animals of the National Institutes of Health. The protocol was approved by the Institutional Animal Care and Use Committee of the University of Washington, Seattle, WA (Protocol: 3108–01). Mice were housed in specific pathogen-free facilities.

***DSG2 transgenic mice:*** Human DSG2 transgenic mice were generated using the bacterial artificial chromosome (BAC) clone CTD-2233I9 (Invitrogen (Carlsbad, CA, USA) containing the human genome region from position 29,054,158 to position 29,143,265 of chromosome 18. This fragment contains the 24 kb DSG2 promoter and 5′ untranslated regions (UTR), the 50.6 kb DSG2 gene, and 14.5 kb of the 3′ UTR. DSG2tg mice contain two copies of the BAC fragment inserted into chromosome 16 [37]. In previous studies we have shown that DSG2 transgenic mice express hDSG2 at a level and in a pattern similar to humans [38].

***Subcutaneous TC1-DSG2^high^.*** Tumors were established by subcutaneous injection of 2 × 10^6^ TC1-DSG2^high^ cells (mixed 1:1 with Matrigel) into the right lower back side. Tumor volumes were measured as described previously [39]. Ad5/3 vectors were injected when tumors reached a volume of ~400 mm^3^.

***Luciferase and GFP in vivo imaging:*** In vivo luciferase imaging was performed on an IVIS Lumina Series II (PerkinElmer, Waltham, MA, USA). Mice were injected i.p. with 15 mg/mL Luciferin in PBS at 150 mg/kg. Five minutes later, animals were transferred to anesthesia induction chamber and animals were induced for 3 min. Animals were then transferred to the in vivo imaging system (IVIS) imaging chamber. Ten minutes after substrate injection, the imaging procedure was started. Exposure times are indicated in the figures.

***GFP immunohistochemistry on tissue sections***. Tissues were either fixed in 10% neutral buffered formalin and processed for paraffin sections. For GFP staining, paraffin sections were subjected to antigen retrieval by antigen unmasking solution (Vector Labs, Burlingame, CA, USA) using a Pascal pressure cooker (DakoCytomation, Carpinteria, CA, USA). For GFP staining, paraffin sections were incubated with monoclonal anti-GFP, 1:400 (Clontech, Mountain View, CA, USA), followed by the Klear Mouse DAB kit (Golden Bridge International Inc., Mukilteo, WA, USA).

***Blood analyses:*** Blood samples were collected into EDTA-coated tubes and analysis was performed on a HemaVet 950FS (Drew Scientific, Waterbury, CT, USA).

***Statistical analysis.*** All results are expressed as mean standard deviation (SD). Student’s t test or 2-way analysis of variance (ANOVA) for multiple testing was applied when applicable. A *p* value of 0.05 was considered significant.

## 3. Results

### 3.1. Ad5/3 Vectors

**Figure 1:**
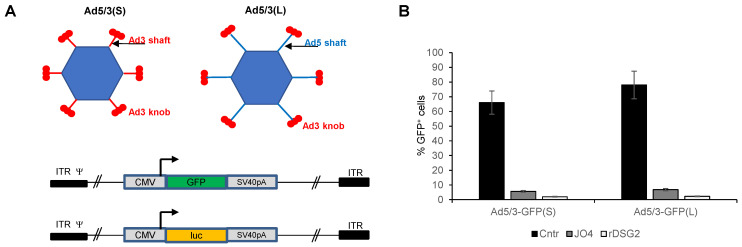
**In vitro transduction studies with Ad5/3(L) and Ad5/3(S) vectors.** (**A**) Vector structure. Upper panel: Vector capsids. Both vectors are based on Ad5. Ad5/3(S) contains the Ad5 fiber tail, the “short” (S) Ad3 fiber shaft and the Ad3 fiber knob. (Ad fibers are homotrimers, which are depicted by three fiber knobs.) Ad5/3(L) contains the original “long” (L) Ad5 fiber shaft and Ad3 fiber knob. Lower panel: Two sets of E1/E3 deleted (first-generation) Ad5/3(S) and Ad5/3(L) vectors were generated containing either a CMV promoter-driven GFP gene (Ad5/3-GFP(S) and Ad5/3-GFP(L) or a CMV promoter-driven firefly luciferase gene (Ad5/3-luc(S) and Ad5/3-luc(L)). SV40pA: SV40 polyadenylation signal. ITR: adenoviral inverted repeat. ψ: adenoviral packaging signal. (**B**) Competition of Ad5/3-GFP(S) and Ad5/3-GFP(L) by recombinant Ad3 fiber knob (JO4) or rDSG2. Colon cancer T84 cells were incubated with 50 μg/mL of proteins for 1 h. Cells were then infected with viruses at an MOI of 100 pfu/cell. Viruses were removed 1 h after infection and GFP expression was analyzed ~20 h later by flow cytometry. Cntr: untransduced control cells.

Ad5/3L vectors contain the Ad5 tail, the “long” (L) Ad5 shaft, and the Ad3 fiber knob. Ad5/3S vectors contain the Ad5 tail, the “short” (S) Ad3 shaft, and Ad3 knob (Figure 1A). All Ad5/3 vectors are E1/E3-deleted. The first pair of “L” and “S” vectors has an identical CMV-GFP expression cassette inserted into the E3 region. The other pair has a CMV-luciferase expression cassette. Vectors were produced in 293 cells and purified by CsCl ultracentrifugation. Vector yields and pfu titers were comparable for all four vectors. To confirm infection of cells through the interaction between fiber knob and DSG2, a competition study was performed (Figure 1B). Transduction of both vectors was inhibited by pre-incubation of cells with recombinant fiber Ad3 knob (JO4) [38], as well as by recombinant DSG2.

### 3.2. Effect of Coagulation Factor X on Transduction with Ad5/3 Vectors

**Figure 2:**
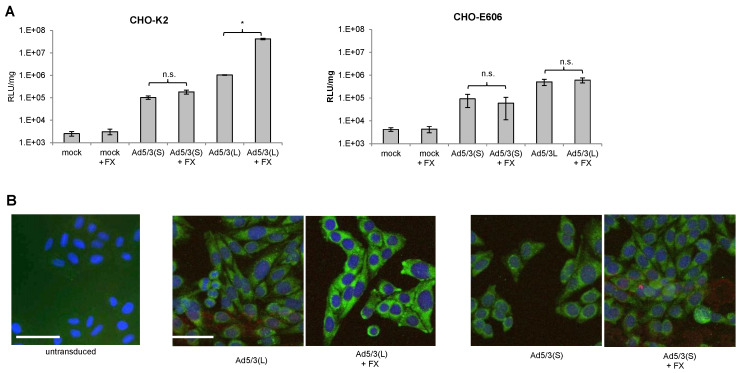
**Effect of coagulation factor X on infection of Ad5/3-luc(S) and Ad5/3-luc (L).** (**A**) Luciferase activity after transduction of CHO-K2 and sulfation-deficient CHO-E606 cells. Viruses were added to cells at an MOI of 150 pfu/cell with or without recombinant factor X (FX) (8 μg/mL), and luciferase expression was measured 20 h later. *n* = 3. * *p* < 0.05; n.s.: not significant. (**B**) Detection of viral particles after infection of CHO cells by immunofluorescence analysis for the viral capsid protein hexon. Six hours after infection of cells with and without FX, cells were fixed and incubated with hexon-specific (green) and cathepsin B-specific (red) antibodies. Untransduced CHO cells were used as a negative control. The scale bar is 20 μm. Representative images are shown.

Ad5 vectors containing the “long” Ad5 fiber shaft and either the Ad5 or Ad35 fiber knob efficiently transduce hepatocytes in vivo, mostly through interactions between the capsid hexon with coagulation factor X (FX) and subsequent uptake by hepatocytes through Heparan-sulfate-proteoglycans (HSPGs) [31,32]. In our previous studies, we found that chimeric Ad5 vectors containing the “short” Ad35 shaft (six shaft motifs) and the Ad35 fiber knob only very inefficiently transduced hepatocytes in mice and non-human primates (NHPs) after intravenous injection [27,40]. We therefore hypothesized that short fiber shafts would interfere with FX-mediated infection of cells. We tested this by using our pair of Ad5/3-luc(S) and Ad5/3-luc(L) vectors on CHO-K2 cells and CHO cells that lack HSPGs (CHO-pgsE-606) (Figure 2). While FX significantly increased Ad5/3-luc(L) transduction of CHO-K2 cells, it had no effect on Ad5/3-luc(S) transduction (Figure 2A). The enhancing effect of FX on Ad5/3-luc(L) was abolished in CHO-psgE-606 cells. Uptake of viral particles was also analyzed based on immunofluorescence of the main capsid protein hexon (Figure 2B). Hexon-specific signals in the cytoplasm were comparable for Ad5/3-luc(L) and Ad5/3-luc(S) in the absence of FX. However, they were stronger for the Ad5/3(L) vectors when the vector was mixed with FX. No colocalization of hexon and lysosomal-specific cathepsin B signals were found indicating that both types of vectors were not transported into lysosomes. Notably, CHO cells do not express human DSG2 and Ad5/3 uptake is mediated through HSPGs, which are the receptors that mediated virus uptake through FX [41].

### 3.3. In Vitro Transduction Studies with Ad5/3 Vectors

**Figure 3:**
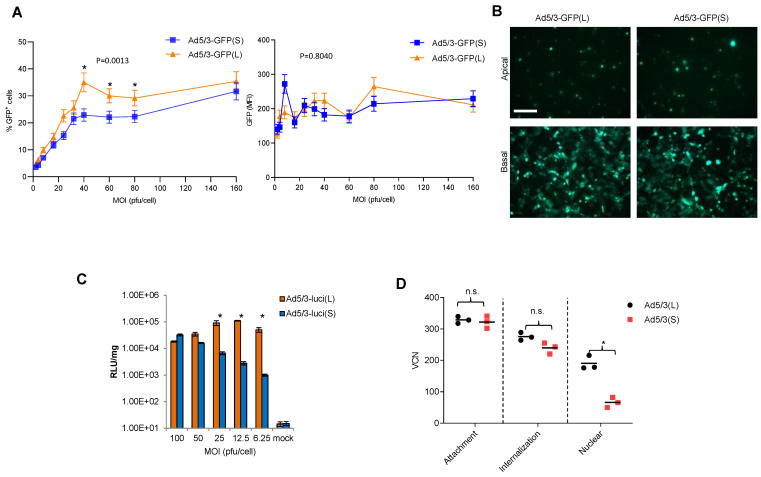
**In vitro transduction studies.** (**A**) T84 cells were infected with Ad5/3-GFP viruses at the indicated MOIs. GFP expression was analyzed 20 h after infection by flow cytometry. The left panel shows the percentage of GFP-positive cells; the right panel shows the mean GFP fluorescence intensity (MFI). *n* = 3. * *p* < 0.05. (**B**) To form tight junctions between T84 cells, 2 × 10^5^ T84 cells were plated into polyester membrane transwell inserts and cultured for 21 days with medium change every 3 days. Ad5/3-GFP(S) and Ad5/3-GFP(L) (MOI 200 pfu/cell) were used to infect cells from the apical or basal sides for 1 h. Viruses were then removed, and fresh medium was added. GFP images were taken 48 h post-infection. The scale bar is 20 μm. (**C**) T84 cells infected with Ad5/3-luc viruses at the indicated MOIs. Luciferase expression was analyzed 20 h after infection and expressed as relative light units (RLU) per mg total protein. *n* = 3. * *p* < 0.05. (**D**) Quantification of viral particles/genomes that were attached, internalized, or transported to the nucleus after infection of HeLa cells with Ad5/3(L) and Ad5/3-GFP(S). Experimental details are shown in Figure S1.

Studies were performed with T84 cells, a human colon cancer cell line that expresses high levels of DSG2 and forms epithelial junctions when cultured in transwells [42]. T84 cells were transduced at increasing MOIs with Ad5/3-GFP(S) and Ad5/3-GFP(L) and GFP expression was measured by flow cytometry 20 h later (Figure 3A). The percentage of GFP-positive cells was significantly higher for Ad5/3-GFP(L) for MOIs of in the range of 40 to 80 pfu/cell, while at the higher MOI (160 pfu/cell), the difference was not significant. No significant differences between the vectors were found in the mean GFP fluorescence at all MOIs analyzed (Figure 3A, right panel). There was also no difference in the number of GFP-positive cells after infection of polarized T84 cells from the apical and basal sides at an MOI of 300 pfu/cell (Figure 3B). Notably, infection from the apical side is inefficient because DSG2 is trapped in lateral epithelial junctions. Transduction studies with Ad5/3-Luc vectors allowed for a more quantitative read-out over a large range of luciferase intensities (Figure 3C). This study confirmed that transduction of T84 cells is less efficient for Ad5/3-luc(S) at low MOIs (6.25, 12.5, 25 pfu/cell), while there is no difference at higher MOIs (50 and 100 pfu/cell).

We then tried to determine at which step of the viral infection process Ad5/3(S) was less efficient than Ad5/3(L) (Figure 3D). Using qPCR for viral genomes, we measured the number of virions that were attached to cells, internalized into the cytoplasm, and imported into the nucleus (Figure S1). In agreement with previous data, there was no significant difference between the two vectors in attachment and internalization [36]. However, the number of vector genomes measured in the nucleus was significantly lower for Ad5/3(S).

### 3.4. In Vivo Transduction of Tumor-Bearing Mice with Ad5/3 Vectors

**Figure 4:**
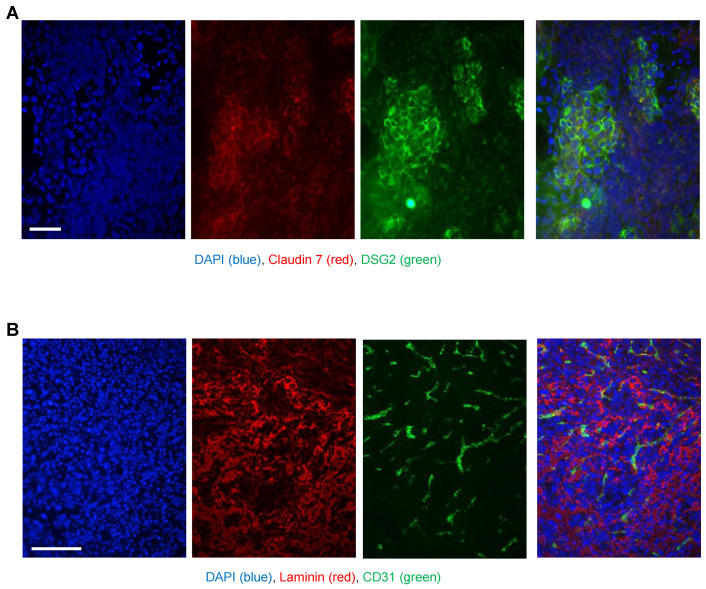
**Immunofluorescence analysis of TC1-DSG2 tumor sections.** Tumors were harvested when they reached a volume of 500–600 mm^3^, embedded in OCT, and sectioned. (**A**) Sections were stained for (human) DSG2 (green) and the epithelial marker claudin 7 (red). Visible are nests of human DSG2-postitive tumor cells embedded in tumor stroma cells (mouse-derived). (**B**) Sections were stained for the extracellular matrix/stroma protein (red) and the endothelial/blood vessel cell marker CD31 (green). The images show a vascularized tumor with typical stroma components.

As an adequate model for biodistribution studies after intravenous Ad5/3 vector injection, we used human DSG2 transgenic mice, and as a control, non-transgenic litter mates. DSG2tg mice contain the human DSG2 locus and express human DSG2 at a level and in a pattern similar to those found for humans and nonhuman primates [37]. We developed a syngeneic tumor cell line (TC1-DSG2) that expressed human DSG2 at a high level. Subcutaneous implantation of TC1-DSG2 cells into the right lower back side resulted in the development of tumors that reached the endpoint volume (1 cm^3^) within 8 weeks after implantation. To characterize TC1-DSG2 tumors, we performed immunofluorescence studies on tumor sections (Figure 4). DSG2-positve tumor nests contained epithelial junctions marked by claudin 7 and were surrounded by mouse-derived stoma cells and extracellular matrix proteins such as laminin. Tumors and tumor stroma contained blood vessels marked by CD31. These features are similar to tumors found in humans. The DSG2tg + TC1 − DSG2^high^ mouse model therefore represents an adequate model for vector biodistribution studies.

#### 3.4.1. In Vivo Analysis of GFP Expression

**Figure 5:**
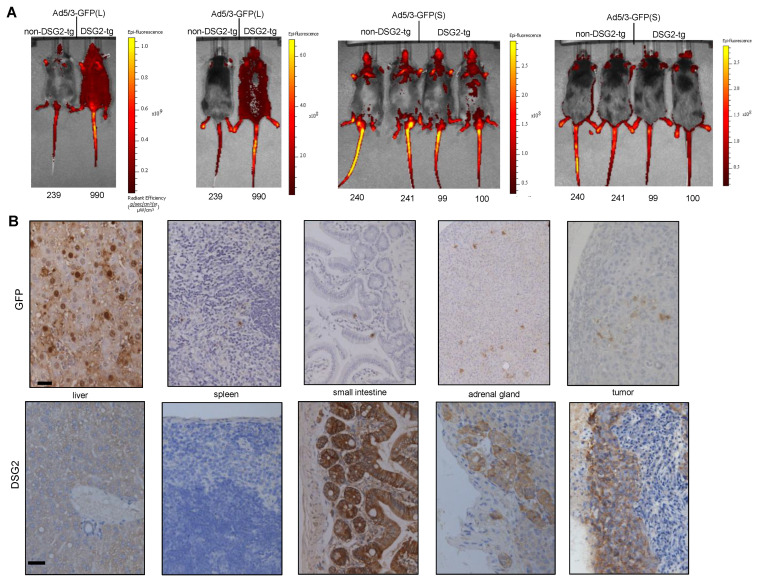
**Biodistribution of GFP expression after intravenous Ad5/3 injection.** (**A**) In vivo GFP imaging. TC1-DSG2^+^ tumor-bearing non-DSG2 transgenic mice (litter mates) and DSG2-transgenic mice were intravenously injected via the tail with pfu of Ad5/3-GFP(L) or Ad5/3-GFP(S). Mice were subjected to imaging 3 days later. Shown are two mice injected with Ad5/3-GFP(L) and four mice injected with Ad5/3-GFP(S) from the font and the back. The color reflects the radian efficiency, the range of which is indicated in the bars to the right of the images. The tag numbers are indicated below the images. (**B**) GFP and DSG2 immunohistochemistry analyses of selected organs and the tumor harvested on day 3 after Ad5/3-GFP (L) injection. GFP staining appears brown. The scale bar is 20 μm. Shown are representative sections of mouse #990. DSG2 staining of tumor sections is shown in Figure 4A.

First, we injected tumor-bearing mice intravenously with Ad5/3-GFP(L) and Ad5/3-GFP(S) and analyzed GFP expression 3 days later by in vivo imaging of animals and immunohistochemistry (IHC) analysis of tissue sections (Figure 5). In vivo imaging showed widespread superficial signals in DSG2tg mice injected with Ad5/3-GFP(L) but not in non-transgenic mice injected with this virus or mice injected with Ad5/3-GFP(S) (Figure 5A). These signals most likely originated from transduced cells in the skin because GFP signals that are deeper in the body cannot be detected by this method. Using this method, it was also difficult to discern tumor-localized signals. More informative were the IHC studies (Figure 5B). GFP signals were found in hepatocytes of liver sections from DSG2tg mice injected with Ad5/3-GFP(L) GFP signals were found on tumor sections of Ad5/3-GFP(L). Sparse GFP signals were also visible in the spleen, intestine, and adrenal gland. When correlated with DSG2 staining of consecutive sections (Figure 5B, lower panels), transduction in the intestine and adrenals appeared to be DSG2-dependent, whereas Ad5/3-GFP(L) transduction of the liver was not. In contrast, GFP signals in Ad5/3-GFP(S)-injected animals were only found in the liver (Figure S3), but not in other organs and the tumor. While ~50% of hepatocytes were positive in Ad5/3-GFP(L) injected animals, only sparse GFP-positive hepatocytes were visible on liver sections from mice that received Ad5/3-GFP(S).

#### 3.4.2. In Vivo Analysis of Luciferase Expression

**Figure 6:**
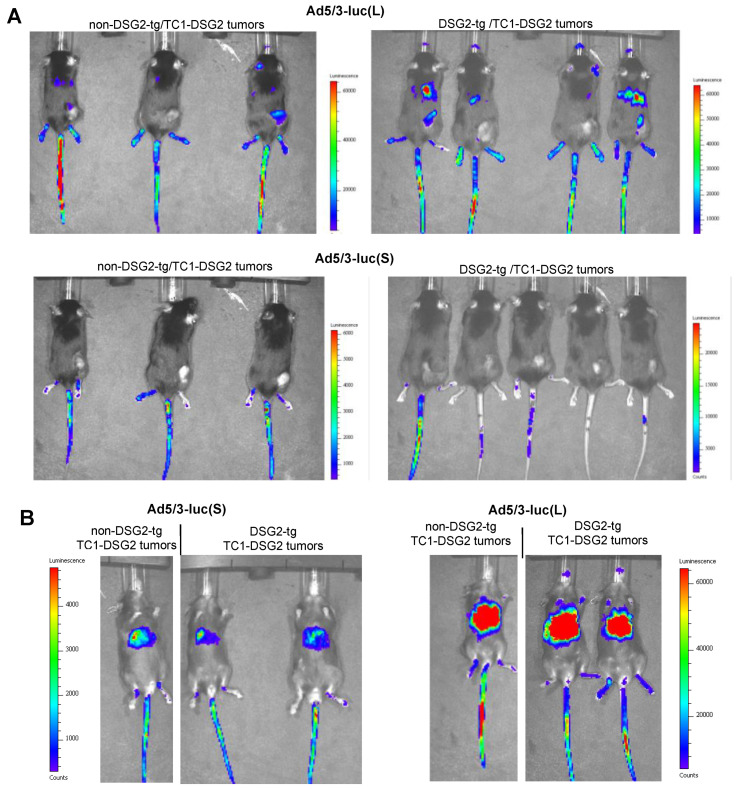
**In vivo imaging for luciferase expression after intravenous Ad5/3 injection.** (**A**) Images taken from the back of mice on day 3 after virus injection. Shown are three non-DSG2-tg/TC1-DSG2 tumors and four DSG2-tg/TC1-DSG2 tumor mice injected with Ad5/3-luc(L) or Ad5/3-luc(S). Tumors are visible at the lower right back side. Strong signals at the injection site (tail veins) are noted. The exposure time was 180 s. (**B**) Images taken from the front of mice.

**Figure 7:**
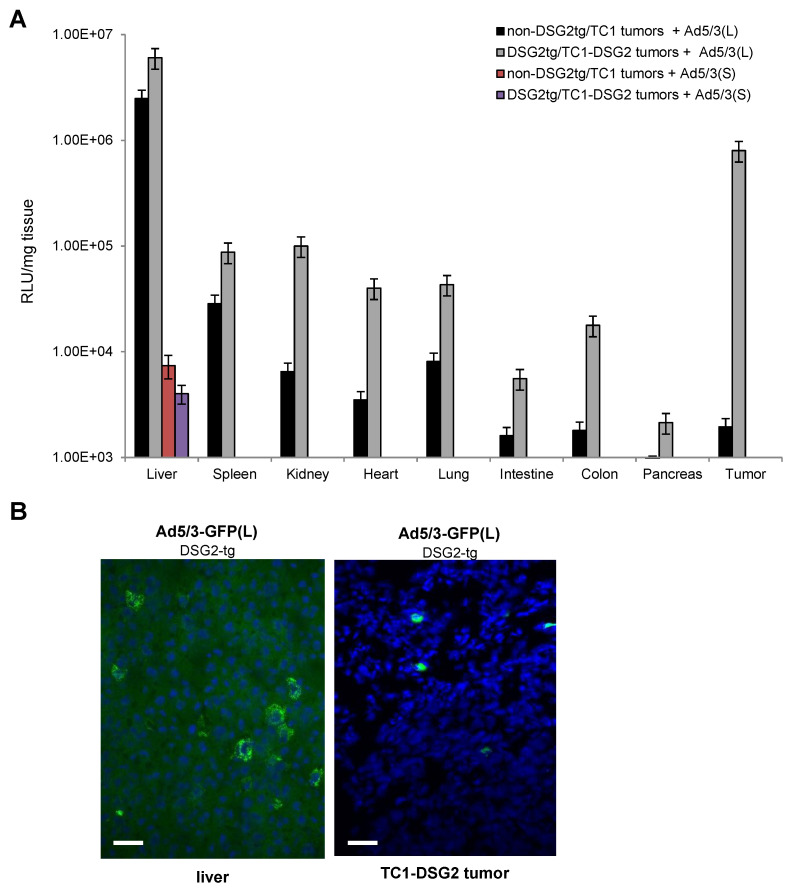
Luciferase activity measured in organs and the tumor harvested on day 3 after intravenous Ad5/3-luc virus injection. (**A**) Summary of luciferase activity data. Shown are data for tumor-bearing DSG2-transgenic and non-transgenic mice injected with the two Ad5/3-luc vectors. Signals from Ad5/3(S)-injected mice were below background, except for the liver. *n* = 3. (**B**) Immunofluorescence staining with a luciferase-specific antibody (FITC—green) of liver and tumor sections from a DSG2-tg mouse injected with Ad5/3(L).

Luciferase in vivo imaging is a more adequate method for biodistribution studies (Figure 6 and Figure S2). Imaging from the back side revealed luciferase luminescence signals in TC1-DSG2 tumors and livers in both DSG2tg and non-DSG2tg mice injected with the long-shafted Ad5-luc(L) vector (Figure 6A and Figure S2A). No signals, except for the injection site in the tail vein, were detected by in vivo imaging for Ad5/3-luc(S) injected animals. Imaging from the front side (Figure 6B and Figure S2B) showed liver-localized signals in Ad5/3-luc(S] animals, however at a much lower intensity than in Ad5/3-luc(L) injected animals. To quantitate luciferase activity, organs and tumors were harvested at day 3 post virus injection, and lysates were subjected to luciferase assay (Figure 7A). Background activity (1 × 10^3^ RLU/mg total protein) established in mock-injected mice was subtracted from all values and RLUs/mg protein were plotted. For the short-shafted Ad5/3-luc(S) vector, luciferase activity was only detectable in the liver of both DSG2tg and non-DSG2tg mice at a level that was 2–3 orders of magnitude lower than liver-localized luciferase activity in Ad5/3-luc(L) injected mice. The greatest difference between DSG2tg and non-DSG2tg mice was, however, found for TC1-DSG2 tumors indicating that the level and accessibility of DSG2 in tumors mediated efficient transduction. Transduction of other tissues was less efficient than tumor transduction. For Ad5/3-luc(L), activity in epithelial tissues (kidney, lung, intestine, colon, pancreas) and heart was higher in DSG2tg mice than in non-DSG2tg mice, indicating DSG2-mediated transduction. In contrast, Ad5/3-luc(L) transduction of liver and spleen seemed to be DSG2-independent. Luciferase signals in the liver and the tumor originated from transduced hepatocytes and tumor cells, respectively, as immunofluorescence studies indicated (Figure 7B).

**Figure 8:**
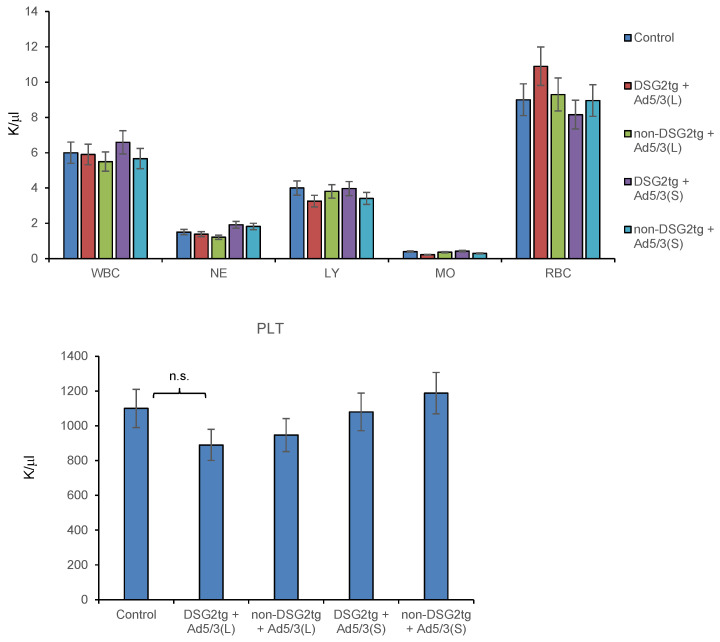
**Hematological analyses of Ad5/3-luc injected mice.** Blood samples were analyzed on day 3 after virus injection. Shown are data for tumor-bearing DSG2-transgenic and non-transgenic mice injected with the two Ad5/3-luc vectors. *n* = 3. WBC–white blood cells, NE–neutrophils, LY–lymphocytes, MO–monocytes, RBC–red blood cells. Platelet (PLT) counts are shown in the lower panel.

Hematological analyses on day 3 after injection of both vectors did not show significant differences between the virus-injected groups and untreated animals (Figure 8).

In summary, intravenous injection of Ad5/3-luc(L) resulted in TC1-DSG2^high^ tumor transduction, however, also in transduction of hepatocytes, most likely via FX. DSG2-mediated Ad5/3-luc(L) transduction of other epithelial tissues was 1–2 orders of magnitude lower than tumor-transduction, likely because DSG2 is not readily accessible in normal epithelial tissues. The Ad5/3-luc(S) vector was nearly inactive in vivo, except for some DSG2-independent liver transduction that was 2–3 orders of magnitude lower than that of Ad5/3-luc(L) injection animals.

## 4. Discussion

This study showed that Ad5/3(L) vectors efficiently transduced DSG2^high^ tumors but not normal epithelial tissues after intravenous injection into DSG2tg mice that express DSG2 in a pattern similar to humans. The basis for preferential tumor transduction is differences in level and accessibility of the target receptor DSG2. While in normal epithelial tissue DSG2 is trapped in epithelial junctions and not accessible to DSG2 ligands such as recombinant Ad3 fiber knobs [38,42], it is expressed at high levels on the surface of tumors, with only a fraction trapped in junctions [42].

The ability of Ad5/3L to target epithelial tumors comes with the caveat that it also transduces hepatocytes in a DSG2-independent manner after intravenous injection into mice. Our in vitro studies indicate that hepatocyte transduction is facilitated by binding of the Ad5 hexon to vitamin K-dependent coagulation factors, which is in agreement with in vivo studies by Koski et al. [43]. Our in vitro studies also show that factor X does not enhance transduction with Ad5/3(S) that contained the same Ad3 fiber knob, but the short Ad3 fiber shaft. This could be due to several mechanisms: (i) the interaction between the hexon Gla-domain and factor X is sterically affected by the short rigid Ad3 fiber, and/or (ii) the high affinity of the Ad3 fibers for DSG2 partially overcomes the Ad5 hexon:FX interaction [30].

Previous studies performed by us [36] and others [44] did not indicate differences in infectivity of Ad5/3(L) and Ad5/3(S), most likely because in these studies relatively high MOIs (>250 pfu/cell) of viruses were used for infection. Here, we performed in vitro transduction studies with lower MOIs (<100 pfu/cell). Under these conditions, transduction measured based on GFP-positive cells or luciferase activity was significantly less efficient for the short-shafted Ad5/3(S) vector. Moreover, in vivo transduction of tumors and tissues was not detectable after intravenous injection into DSG2-transgenic mice, except for the liver where luciferase signals were 2–3 orders of magnitude lower compared Ad5/3(L), supporting our hypothesis that Ad5/3(S) only inefficiently transduces hepatocytes.

The DSG2-dependent infectivity of Ad5/3(S) could be affected at several stages of the virus infection process, including virus attachment, internalization, endosome release, intracellular trafficking to the nucleus, and import of the viral genome into the nucleus. Our data indicate that after internalization, intracellular trafficking of Ad5/3(S) is impaired. Based on our in vitro studies with long- and short-shafted Ad5 vectors [26], we hypothesize that this is due to inefficient release from endosomes and/or retrograde transport of particles to the cell surface. Release from endosomes is influenced by the penton base RGD motif-integrin α_v_/β_3/5_ interaction, either through mediating pH changes within endosomes that lead to membrane permeabilization, or through integrin-mediated intracellular signaling [45]. In wild-type Ad3 virus, the protruding penton RGD loop is longer than the RGD loop in Ad5 (Figure S4). This could imply that in Ad5/3(S), the spatial constellation of Ad3 knob/DSG2 and RGD/integrins is not optimal for endosome release. Replacing the Ad5 RGD loop in Ad5/3(S) with that of Ad3 could prove this hypothesis. This hypothesis is also supported by publications that the Ad3 vectors (containing the Ad3 fiber knob and shaft and Ad3 penton) efficiently infect cells [3,46] and trigger oncolysis in vivo [19,47]. We speculate that inefficient intracellular trafficking of Ad5/3(S) is also responsible for the poor TC1-DSG2 tumor transduction in vivo.

An approach to enhance in vivo transduction of target cells after intravenous Ad injection is to increase the affinity of the fiber knob to the target receptor by introducing mutations to the fiber knob [48]. In the case of affinity enhanced Ad5/35(S) vectors, we demonstrate, in a mouse model with pre-established CD46^high^ liver metastases, better tumor transduction without significant liver transduction [21]. Furthermore, such an Ad5/35 vector enabled us to transduce mobilized hematopoietic stem cells (HSCs) in mice [49] and non-human primates [50], which was very inefficient with an Ad5/35(S) vector without enhanced affinity to CD46. Recently, we showed that a short-shafted Ad5/3 vector containing Ad3 fiber knobs with greatly increased affinity to DSG2 also efficiently transduced mobilized hematopoietic stem cells (HSCs) in NHPs [51,52]. We speculate that the high avidity, together with the high DSG2 receptor density and accessibility of DSG2 on HSCs, could compensate for the deficiencies of Ad5/3(S) vectors found in this study.

## 5. Conclusions

This is the first biodistribution study of Ad5/3(L), a vector platform that is used clinically in oncolytic virotherapy in an adequate DSG2tg animal model. It shows tumor targeting with minimal transduction of normal epithelial tissues after intravenous injection. However, Ad5/3(L) also efficiently transduced hepatocytes implying that careful monitoring of liver inflammation is recommended in studies with this vector platform. Ad5/3(S) vectors showed minimal liver transduction however failed to transduce DSG2^high^ tumors. Further modifications of Ad5/3(S) in the Ad5 penton base or Ad3 fiber knob are probably required to compensate for the lower infectivity of these vectors, which is most likely due to relatively inefficient intracellular trafficking of virions.

## Data Availability

Not applicable.

## Supplementary Materials

The following supporting information can be downloaded at: https://www.mdpi.com/article/10.3390/genes13112056/s1, Figure S1: Workflow for analysis of localization of viral particles/genomes (attached to the membrane, internalized into the cytoplasma, and imported into the nucleus.) Figure S2: In vivo images for luciferase expression after intravenous Ad5/3 injection taken at shorter exposure times. Figure S3: Amino acid alignment of penton base of Ad5 and Ad3. Figure S4: Amino acid alignment of penton base of Ad5 and Ad3. The RGD loop is underlined. Ad3 penton base accession#: ABB17799; Ad5 penton base accession#: AP000206.
