# Supplementary material for: Role of Fiber Shaft Length in Tumor Targeting with Ad5/3 Vectors
Source: Genes (Basel). 2022 Nov 7;13(11):2056. doi: 10.3390/genes13112056 (PMC9690795; doi:10.3390/genes13112056)
Supplement: Supplementary file 1 [file genes-13-02056-s001.zip › genes-1979609-supplementary.pdf]

Treat HeLa cells with Versen, wash, leave on ice for 1h

↓

Incubate with Ad (600vp/cell) in adhesion buffer for 1h

↓

Wash 2x with 500μl PBS → cell pellet ⟶ attachment

↓

Resuspend cells in 100μl adhesion buffer, incubated at 37°C for 30 min. Dilute threefold with cold 0.05% trypsin–0.5 mM EDTA solution and incubated at 37°C for an additional 10 min

↓

Wash 2x with 500μl PBS → cell pellet ⟶ internalization

↓

Resuspend with 1x pre-extraction* buffer (100μl/1M cell), leave on ice for 30min →Vortex vigorously for 10 seconds for every 10min → centrifuge for 1 minute at 13,000 rpm. Carefully remove the cytoplasmic extract from the nuclear pellet.

*abcam nuclear extraction kit

↓

Wash 1x with 500μl PBS→cell pellet ⟶ nuclear localization

Fig.S1. Workflow for analysis of localization of viral particles/genomes (attached to the membrane, internalized into the cytoplasma, and imported into the nucleus.)

A

back – 60 s

No virus
DSG2-tg
TC1-DSG2$^+$ tumors

Ad5/3-luc(L)
non-DSG2-tg
TC1 tumors

Ad5/3-luc(L)
DSG2-tg
TC1-DSG2$^+$ tumors

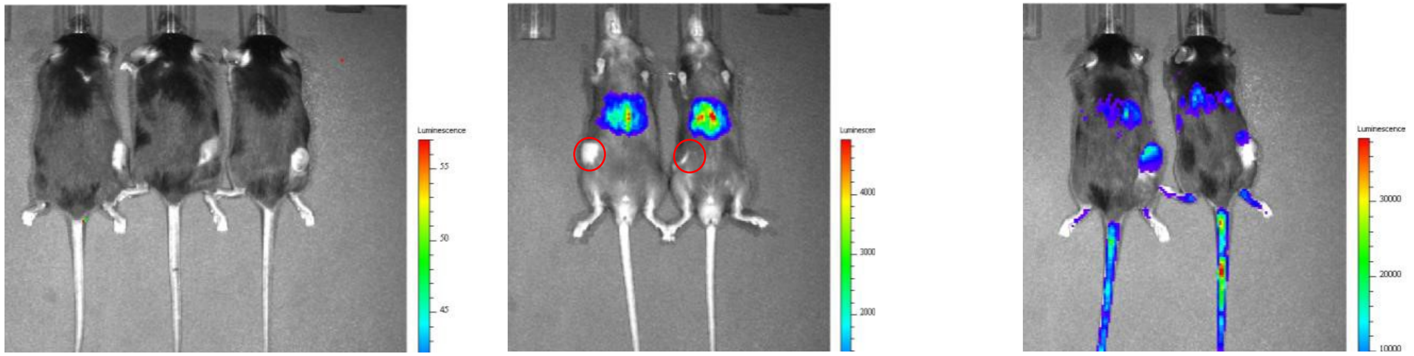

B

front – 0.1 s

Ad5/3-luc(L)
non-DSG2-tg
TC1-DSG2$^+$ tumors

Ad5/3-luc(L)
DSG2-tg
TC1-DSG2$^+$ tumors

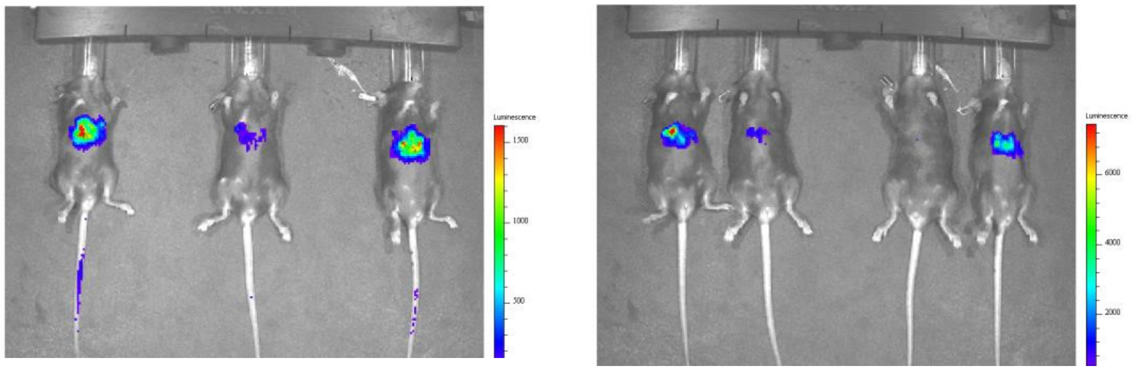

Fig.S2. ***In vivo*** **images for luciferase expression after intravenous Ad5/3 injection taken at shorter exposure times. A)** Shown are images (with 60 seconds of exposure) of three control mice that were not injected with Ad, two HDAd5/3(L) injected mice bearing TC1 tumors that do not express DSG2, and two TC1-DSG2-tumor-bearing DSG2-transgenic mice injected with Ad5/3luc(L), **B)** Shown are front images (with 0.1 seconds of exposure) of three tumor-bearing non-DSG2 transgenic mice and four DSG2 transgenic mice injected with Ad5/3luc(L).

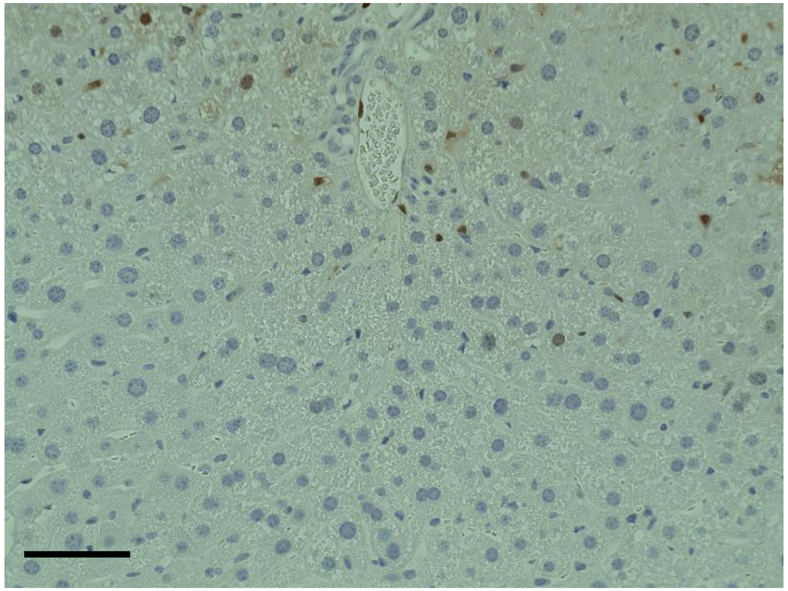

Fig.S3. GFP immunohistochemistry analysis of a liver (mouse #100) at day 3 after Ad5/3-GFP(S) injection into DSG2tg mice. GFP staining appears brown. The scale bar is 20μm.

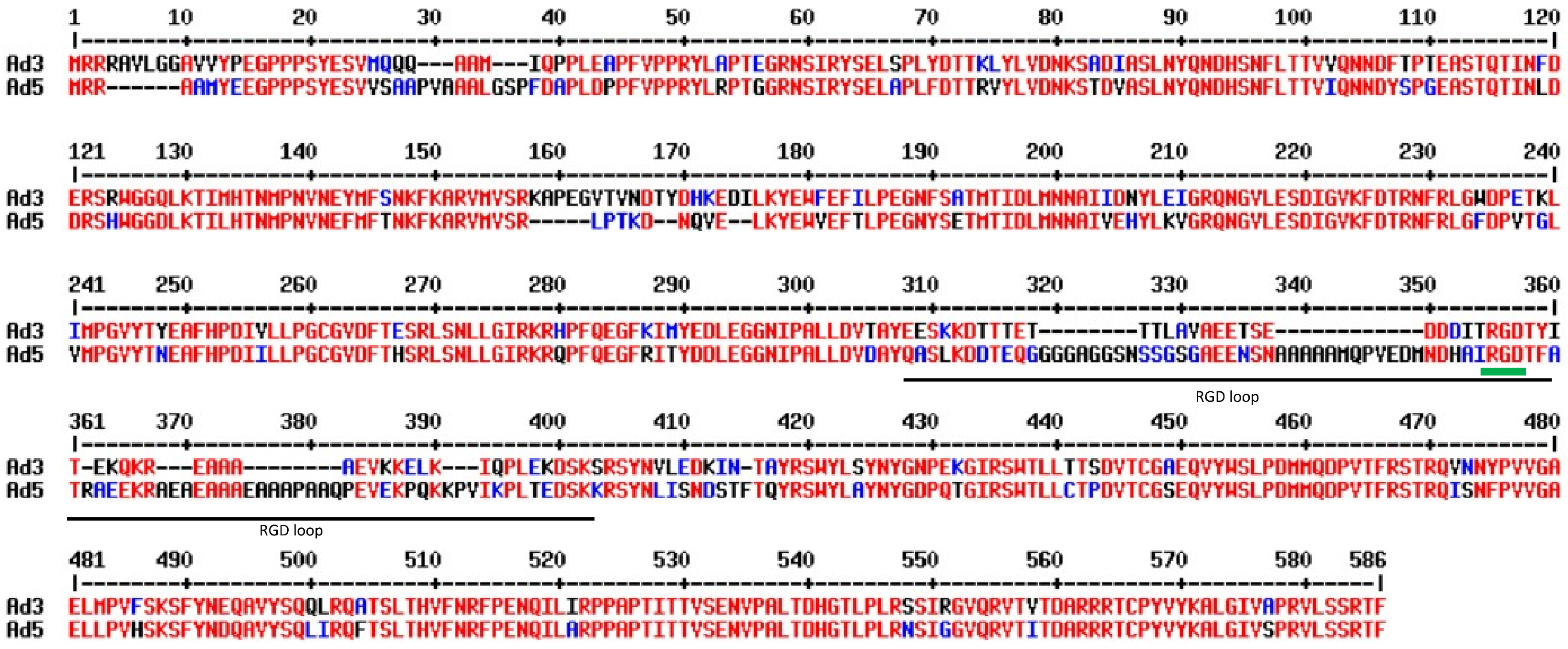

Fig.S4. Amino acid alignment of penton base of Ad5 and Ad3. The RGD loop is underlined. Ad3 penton base accession#: ABB17799; Ad5 penton base accession#: AP000206
